# Examining the effects of HIV self-testing compared to standard HIV testing services: a systematic review and meta-analysis

**DOI:** 10.7448/IAS.20.1.21594

**Published:** 2017-05-15

**Authors:** Cheryl C Johnson, Caitlin Kennedy, Virginia Fonner, Nandi Siegfried, Carmen Figueroa, Shona Dalal, Anita Sands, Rachel Baggaley

**Affiliations:** ^a^ Department of HIV, World Health Organization, Geneva, Switzerland; ^b^ Social and Behavioral Interventions Program, Department of International Health, Johns Hopkins University Bloomberg School of Public Health, Baltimore, MD, USA; ^c^ Division of Global and Community Health, Department of Psychiatry and Behavioral Sciences, Medical University of South Carolina, Charleston, SC, USA; ^d^ Independent Clinical Epidemiologist, Cape Town, South Africa; ^e^ Department of Essential Medicines and Health Products, World Health Organization, Geneva, Switzerland

**Keywords:** HIV/AIDS, HIV test, HIV self-test, public health

## Abstract

**Introduction**: HIV self-testing (HIVST) is a discreet and convenient way to reach people with HIV who do not know their status, including many who may not otherwise test. To inform World Health Organization (WHO) guidance, we assessed the effect of HIVST on uptake and frequency of testing, as well as identification of HIV-positive persons, linkage to care, social harm, and risk behaviour.

**Methods**: We systematically searched for studies comparing HIVST to standard HIV testing until 1 June 2016. Meta-analyses of studies reporting comparable outcomes were conducted using a random-effects model for relative risks (RR) and 95% confidence intervals. The quality of evidence was evaluated using GRADE.

**Results**: After screening 638 citations, we identified five randomized controlled trials (RCTs) comparing HIVST to standard HIV testing services among 4,145 total participants from four countries. All offered free oral-fluid rapid tests for HIVST and were among men. Meta-analysis of three RCTs showed HIVST doubled uptake of testing among men (RR = 2.12; 95% CI: 1.51, 2.98). Meta-analysis of two RCTs among men who have sex with men showed frequency of testing nearly doubled (Rate ratio = 1.88; 95% CI: 1.17; 3.01), resulting in two more tests in a 12–15-month period (Mean difference = 2.13; 95% CI: 1.59, 2.66). Meta-analysis of two RCTs showed HIVST also doubled the likelihood of an HIV-positive diagnosis (RR = 2.02; 95% CI: 0.37, 10.76, 5.32). Across all RCTs, there was no indication of harm attributable to HIVST and potential increases in risk-taking behaviour appeared to be minimal.

**Conclusions**: HIVST is associated with increased uptake and frequency of testing in RCTs. Such increases, particularly among those at risk who may not otherwise test, will likely identify more HIV-positive individuals as compared to standard testing services alone. However, further research on how to support linkage to confirmatory testing, prevention, treatment and care services is needed. WHO now recommends HIVST as an additional HIV testing approach.

## Introduction

Global scale-up of HIV testing services (HTS) has been significant. From 2010 to 2014, more than 600 million people received HTS in 122 low- and middle-income countries [1]. This expansion has been made possible through the widespread introduction of provider-initiated testing and counselling and an array of community-based approaches which are now considered the standard of care [2]. Despite this, approximately 40% of all HIV infections are undiagnosed worldwide [3] and countries are seeking ways to increase the number of people who know their HIV status to achieve the first of the United Nation’s 90-90-90 HIV testing and treatment goals – diagnosis of 90% of all people with HIV by 2020 [4].

HIV self-testing (HIVST) has been proposed as an approach to reach people who are not accessing existing HTS, such as men, young people, and key populations (i.e. people who inject drugs, men who have sex with men, sex workers, and transgender people). HIVST refers specifically to a process in which a person collects his or her specimen (oral fluid or blood) and performs a test and interprets the result, often in private or with someone they trust [2].

Several observational studies [5–17] and systematic reviews [18–21] have shown HIVST can be performed accurately and is an acceptable and feasible testing approach in a variety of contexts; including among populations at ongoing HIV risk and those who may not otherwise test. As a discreet, convenient and empowering approach, many well-documented barriers to standard HTS, such as long-lines, services offered at inconvenient times, fear of stigma and lack of confidentiality [22], can be addressed by HIVST [18,23–26].

To assess the potential effects of HIVST compared to standard HTS, that is, facility- or community-based approaches, we conducted a systematic review. Our objective was to assess the effects of HIVST on uptake and frequency of HIV testing, diagnosis of people with HIV, linkage to prevention and care, risk behaviour, social harm or other adverse events, compared to standard HTS. Review findings were then used to help determine whether HIVST should be recommended as an additional HTS approach in WHO guidelines.

## Methods

This review followed guidance from the Cochrane Collaboration [27] and the PRISMA statement for the reporting of systematic reviews and meta-analyses. The review protocol and the full quality assessment are available in Appendix 1–2 (Supplemental material).

### Search strategy and inclusion criteria

We searched five electronic databases PubMed, CINAHL, PsycINFO, Sociological Abstracts, and EMBASE through 1 June 2016 for peer-reviewed articles. We also searched the following conference databases for abstracts: International AIDS Conference (IAC), International AIDS Society Conference on HIV Pathogenesis, Treatment, and Prevention (IAS), and Conference on Retroviruses and Opportunistic Infections (CROI). IAC and IAS conference abstracts were searched for all available years (2001–2015). For CROI, only recent conferences (2014–2016) were searched as past conferences were inaccessible. Secondary reference searching was conducted on all studies included in the review as well as on previously published reviews. We also contacted experts to identify additional studies, specifically abstracts being presented at the 2016 IAC, and reviewed databases listing ongoing RCTs through clinicaltrials.gov, WHO International Clinical Trials Registry Platform, and Pan African Clinical Trials Registry.

The search strategy was adapted for entry into all computer databases using key terms “HIV”, “self-test”, and “home test” (Appendix 1 (Supplemental material)). To search HIV-related conference abstracts, only terms for self-testing were used because search functions were limited. No language or geographic limitations were placed on the search.

Two reviewers (CK and VF) screened studies. The first reviewer identified study titles and abstracts meeting the inclusion criteria. The second reviewer evaluated the application of screening criteria and approved selected studies. Disagreements between reviewers were resolved through discussion and consensus. CJ, VF, CK also contacted all authors of studies included in the review to collect additional information about each study.

To be included, studies needed to directly compare HIVST to HTS by a provider in either a facility or community setting (defined as standard HTS) and report on one or more of the following outcomes: (1) uptake of HIV testing (e.g. the number of participants who tested for HIV in the study period); (2) frequency of HIV testing (e.g. the number of times a participant tested for HIV in the study period); (3) social harm/adverse events (defined as any undesirable experience, or intended or unintended harm associated with HIV self-testing); (4) HIV positivity (e.g. the proportion of people with a reactive self-test who received confirmatory HTS and were diagnosed HIV positive); (5) proportion of people linked to confirmatory testing, clinical assessment or treatment and/or measurement of CD4 or viral load among those diagnosed HIV positive; (6) linkage to prevention services following nonreactive self-test result; or (7) sexual risk behaviour (measured as report of condomless sex, sexual transmitted infections (STIs) or number of sexual partners).

Additionally, we also searched for the full-text publication of any abstract included in the review as of 15 March 2017 to check for updates to previous reports.

### Data analysis

Data were extracted independently by four reviewers using standardized extraction forms. Risk of bias was assessed according to guidance by the Cochrane Collaboration and determined by CK, VF, NS, and CJ [27]. Where multiple studies reported the same or comparable outcomes, meta-analyses were conducted using random-effects models to combine relative risks for dichotomous data, mean differences for continuous data, or rate ratios for frequency data, with 95% confidence intervals using REVMAN 5.3.5.

### Quality assessment

GRADE methodology was used to assess and appraise the quality of evidence for each outcome across all studies, and included an evaluation of the risk of bias, imprecision, indirectness, and inconsistency, and other considerations including publication bias [28] (Appendix 2 (Supplemental material)).

## Results

**Figure 1.**
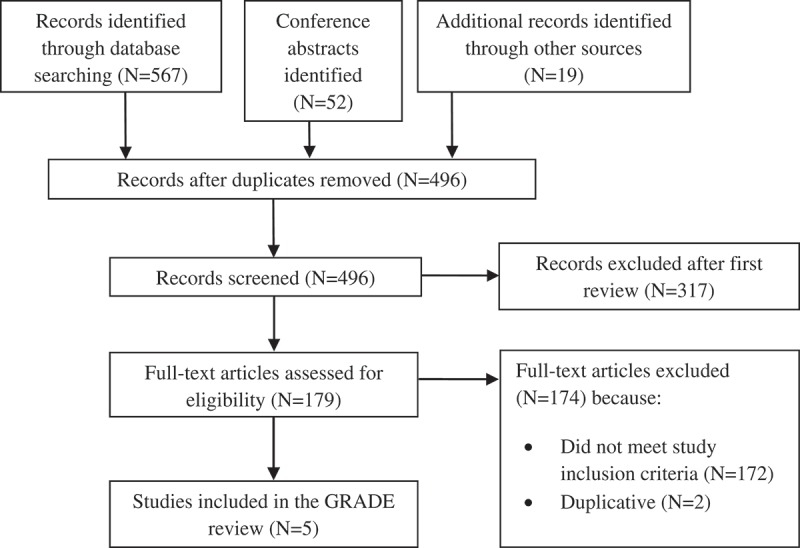
Study selection.

The searches yielded 638 citations, which after screening resulted in five eligible RCTs (Figure 1).

### Study characteristics

All five RCTs were published between 2015 and 2017. Three were full-text manuscripts [29–31], one of which was in press [31,32], and two were conference abstracts [33,34]. These studies included a total of 4,145 individuals (range: 230–2523). The largest study was among 1410 pregnant women and 1113 of their locatable male partners in Kenya [33]. All RCTs reported outcomes among men: two took place in Kenya where women delivered HIVST to their male partners [30,33] and the remainder were among men who have sex with men (MSM) in Australia [29], Hong Kong SAR [31,32], and the United States [34]. Table 1 summarizes the study characteristics.

**Table 1.** Summary of included study characteristics (n = 5)

Author and location	Population	Study design and intervention	Test kit	Type of support
Gichangi et al., 2016 [33] Kenya	Pregnant women (n = 1410); Male partners of pregnant women (n = 1113) Pregnant women (>18 years of age) attending their first antenatal clinic visit who believed they were not at risk of IPV and had a male partner with unknown or HIV-negative status.	RCT: Women randomized (1:3) to one of three groups: (1) receive 2 HIVST kits and encouragement to distribute a kit to their male partner (intervention); (2) receive standard letter to invite male partner for HIV testing alone or as a couple at clinic (standard of care); or (3) receive a referral card stating importance of male partner testing in prevention-of-mother-to-child-transmission (control). Follow-up was completed at the end of the study at three months.	OraQuick	Directly Assisted: Provided women an HIVST kit which included instructions-for-use, a demonstration on how to use the HIVST kit and interpret the results correctly. Also provided instruction on how to encourage their male partner to test and how to handle their partners in case of a positive result.
Jamil et al., 2017 [29] Australia	High-risk MSM (n = 362) HIV-negative men >18 years of age who could speak or write in the English language reporting >5 partners and CAI in past 3-months.	RCT: Men were randomized (1:1) to either free HIVST or standard facility-based testing. Men in the HIVST group received 4 kits; participants could request up to 12-kits per year free of charge. Kits were picked up at study site or mailed to participants. In both groups, men completed a tablet-based questionnaire at enrolment and subsequent online surveys every 3 months. Participants who did not respond were sent reminders by phone call, SMS or email. Study was completed at 12 months.	OraQuick	Unassisted: Provided HIVST kit with manufacturer instructions, as well as a video link and 24 hr hotline. HIVST kits were also labelled with stickers with local information and resources to access support and for emergencies
Katz et al., 2015 [34] USA	High-risk MSM (n = 230) HIV-negative men >18 years of age who could speak English and had a stable home or mailing address reporting >1 event of CAI with partners of discordant or unknown HIV status, a STI, methamphetamine/popper use, or ≥10 male oral or anal sex partners in the past year	RCT: Men were randomized (1:1) to free HIVST or to standard facility-based testing. All participants were told quarterly HIV testing is recommended, informed about acute HIV infection, given a calendar marked with test dates and were offered reminders to test. All participants were asked to complete quarterly online surveys. Those in the standard care group, completed questionnaires reporting the date and location of HIV testing, reasons for testing, and interval sexual history and substance use. Those in the HIVST group were given a HIVST kit and could receive kits on site or by mail upon request. Men in the HIVST group had unlimited access to HIVST kits, but could not receive more than 1-kit per month. Study was completed at 15 months.	OraQuick	Directly Assisted: Provided HIVST kit with manufacturer instructions and a face-to-face demonstration on how to use the test and included pre-test information, and post-test counselling materials were also provided in-person Also provided a list of local HIV/AIDS and related resources and condoms, and a 24-hr telephone hotline for counselling and technical support
Masters et al., 2016 [30] Kenya	Pregnant or post-partum women (n = 600) Women (18–39 years of age) presenting at post-partum or antenatal care who had a male partner with unknown or known HIV-negative status and did not report being at risk of IPV.	RCT: Women were randomized (1:1) to (1) receive two HIVST kits and encouragement to distribute a kit to their male partner or (2) to receive referral vouchers inviting their male partner for HIV testing alone or as a couple. In both groups, women were provided messages to encourage their male partner to test for HIV. Follow-up was sought every month and at the end of the study at three months.	OraQuick	Directly Assisted: Provided women an HIVST kit with manufacturer instructions and an in-person demonstration on how to use the HIVST kit correctly. Women also received instruction on how to encourage their male partner to test.
Wang et al., 2016 [31,32] Hong Kong SAR, China	MSM (n = 430) Chinese-speaking HIV-negative men > 18 years of age who had not tested for HIV in the past 6-months and had access to online live-chat applications in Hong Kong with no intention to move in the next 6-months.	RCT: Men were randomized (1:1) to (1) HIVST including a free test kit by mail, a video promoting testing, an instructional video on HIVST, HIVST motivational interviewing by phone, and online live-chat pre- and post-testing counselling or (2) to standard HIV testing including a video promoting testing and encouragement to test for HIV. Three surveys were also conducted at baseline, midline at 6-months the study end. Those completing all three surveys received a supermarket coupon in the mail worth HK$50 (US$8).	OraQuick	Unassisted: Provided HIVST kit with manufacturer instructions, plus access to motivational interviewing by telephone and pre- and post-test counselling by nurses through live online chat systems (e.g. Line, Whats App, Skype) who also observed individuals self-testing.

IPV: intimate partner violence; RCT: randomized controlled trial; HIVST: HIV self-test; CAI: condomless anal intercourse; STI: sexually transmitted infections; MSM: men who have sex with men.

All studies offered free oral HIVST kits with the manufacturer’s instructions for use, but differed in terms of the number of kits and the level of assistance provided. In order to encourage quarterly testing, in the United States and Australia, MSM had continuous access to HIVST kits [29,34], and in Australia, participants received four HIVST kits at enrolment. In Kenya, women were provided with two HIVST kits at enrolment (one for them and one for their male partner) [30,33]. In Hong Kong SAR, MSM were provided with only one HIVST kit at enrolment [31,32].

HIVST can be delivered with direct assistance, such as an in-person demonstration on how to self-test, or unassisted using either manufacture instructions for use alone. In addition, other support tools such as telephone hotlines, videos or messaging services may also be provided [2]. Two RCTs [29,31,32] provided unassisted HIVST, but in addition to the test kit participants had access an informational video; and one RCT, also provided motivational interviewing via telephone and counselling through online live-chat services [31,32]. The remainder provided an in-person demonstration on how to self-test (direct assistance) [30,33,34]; two of which provided women a demonstration so they could show their male partners how to self-test [30,33].

### Uptake of HIV testing

**Figure 2.**
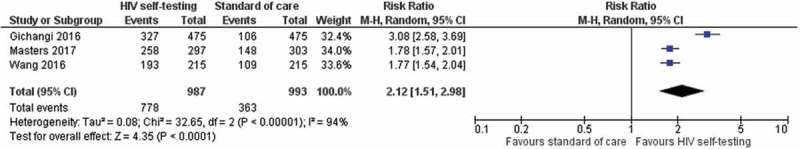
Uptake of HIV testing over three and six month periods among male partners of pregnant women and men who have sex with men.

Three RCTs [30–33] reported on uptake of HIV testing (Table 2). A meta-analysis showed moderate-quality evidence that HIVST doubled the uptake of HIV testing compared to standard HTS (RR = 2.12; 95% CI: 1.51, 2.98; Tau^2^ = 0.08; Chi^2^ = 32.88, df = 2 (p = 0.001; I^2^ = 94%)) (Figure 2). The high level of statistical heterogeneity was driven by the Gichangi and colleagues RCT [33], which measured uptake among men who had accepted some form of HIV testing and did not include those who declined testing. Since the estimate of effects was beneficial for all three RCTs, we did not downgrade for inconsistency. Two RCTs [30,33], where women delivered HIVST to their male partners, also reported HIVST increased uptake of couples testing compared to standard HTS, with moderate-quality evidence (Table 2).

**Table 2.** Summary of select study outcomes (n = 5)

			Uptake of overall HIV testing				Uptake of couples HIV testing				Mean test frequency		HIV positivity (%)	
Author /Year	Country	Population	Standard HTS	n (%)	HIVST	n (%)	Standard HTS	n (%)	HIVST	n (%)	Standard HTS	HIVST	Standard HTS	HIVST
Gichangi et al., 2016 [33]	Kenya	Male partners of pregnant women	471	132 (28)	472	373 (79)	471	106 (22.5)	472	323 (68.4)	N/A	N/A	N/A	N/A
Wang et al., 2016 [31,32]	Hong Kong SAR, China	MSM	215	109 (50.6)	215	193 (89.7)	N/A	N/A	N/A	N/A	N/A	N/A	N/A	N/A
Masters et al., 2016 [30]	Kenya	Male partners of pregnant women	303	148 (48.8)	297	258 (86.7)	303	95 (31.3)	297	214 (72)	N/A	N/A	4 (1.32)	8 (2.7)
Katz et al., 2015 [34]	United States	MSM	114	N/A	116	N/A	N/A	N/A	N/A	N/A	3.5	5.3	2(1∙8)	4 (3.4)
Jamil et al., 2017 [29]	Australia	MSM	165	N/A	178	N/A	N/A	N/A	N/A	N/A	1.9	4.0	N/A	N/A

HIVST: HIV self-test; HTS: HIV testing services; MSM: men who have sex with men; NA: not applicable.

There was low-quality evidence that HIVST resulted in greater HIV testing uptake among young MSM in Hong Kong SAR (18–25 years of age), including both recent and non-recent testers compared to standard HTS (Young MSM: RR = 1.79; 95% CI: 1.43, 2.24; Recent testers: RR = 1.75; 95% CI: 1.46, 2.08; Non-recent testers: RR = 2.22; 95% CI: 1.61; 3.08) [31,32]. In this same study, MSM who reported condomless anal intercourse at baseline were more likely to test if they were in the HIVST group compared to if they were in the standard testing group (RR = 1.75; 95% CI: 1.26, 1.81) [31,32].

### Frequency of HIV testing

**Figure 3.**
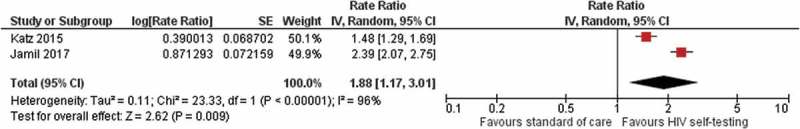
Rate ratio of frequency of testing in a 12–15-month period among men who have sex with men.

**Figure 4.**
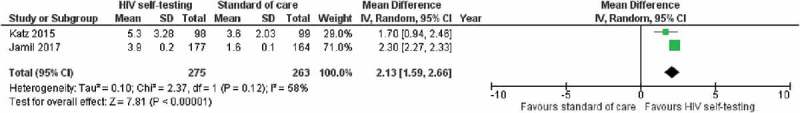
Frequency of HIV testing measured by the mean number of tests in a 12–15-month period among men who have sex with men.

Two RCTs [29,34] in this review, both among MSM, reported on the frequency of HIV testing. Meta-analysis showed there was low-quality evidence that HIVST nearly doubled testing frequency compared to facility-based testing (Rate ratio = 1.88; 95% CI: 1.17; 3.01; Tau^2^ = 0.11, Chi^2^ = 23.33, df = 1 (p < 0.0001), I^2^ = 96%) (Figure 3) and resulted in two more HIV tests in a 12–15-month period than those receiving standard facility-based HTS (Mean difference = 2.13; 95% CI: 1.59, 2.66; Tau^2^ = 0.10; Chi^2^ = 2.37, df = 1 (p = 0∙12), I^2^ = 58%) (Figure 4) [29,34]. In Australia, there was very low-quality evidence that HIVST substantially increased the frequency of testing among non-recent testers compared to standard facility-based HIV testing at 12 months (Rate ratio = 5.54; 95% CI: 3.15, 9.74)[29] (Table 3).

**Table 3.** Summary of study outcomes on uptake and frequency of HIV testing among recent and non-recent testers among men who have sex with men (n = 2)

			Standard HIV testing			HIVST		
Author /Year	Country	Population	Standard HTS	% Uptake	Mean Test Frequency	HIVST	% Uptake	Mean Test Frequency
Wang et al., 2016 [31,32]	Hong Kong SAR, China	MSM Recent tester (>4 tests in 3 years)	30	22(73.3)	NA	24	23(95.8)	NA
		MSM Non-recent tester (1–3 tests in 3 years)	114	61(53.5)	NA	121	113(93.4)	NA
		MSM Non-recent tester (0 tests in 3 years)	71	26(36.6)	NA	70	57(81.4)	NA
Jamil et al., 2017[29]	Australia	MSM Recent tester (tested ≤ 2 years)	141	NA	2.1	148	NA	4.2
		MSM Non-recent tester (tested > 2 years)	24	NA	0.7	30	NA	2.9

NA: not applicable; HTS: HIV testing services; MSM: men who have sex with men.

### HIV positivity

**Figure 5.**
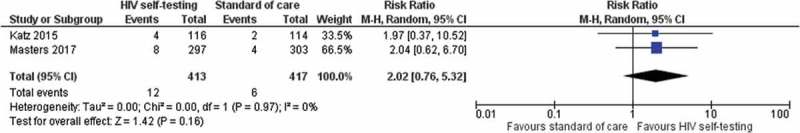
HIV positivity measured by proportion of people reporting an HIV-positive diagnosis.

Two RCTs [30,34] reported on HIV positivity following HIV testing. Meta-analysis showed there was very low-quality evidence that HIVST doubled the likelihood of an HIV-positive diagnosis compared to those using standard testing alone (RR = 2.02; 95% CI: 0.37, 10.76, 5.32) (Figure 5).

### Linkage to care

One RCT in Kenya [30], with very low-quality evidence, reported on linkage to care. In the study, women reported that 25% (n = 2/8) of their male partners in the HIVST group linked to confirmatory testing at 3-month follow-up. Following confirmatory testing, both men were reportedly confirmed HIV positive and then linked to care. In the control group, women reported that all four male partners who were diagnosed HIV positive linked to care.

### Risk behaviour

Two RCTs [31,32,34] reported on risk-taking behaviours. In the United States, there was very low-quality evidence showing that MSM in the HIVST group did not increase condomless anal intercourse compared to those undergoing facility-based HTS (RR = 0.94: 95% CI: 0.55, 1.61) [34]. In this same study, there was very low-quality evidence that men in the HIVST group acquired fewer STIs than those in the standard HTS group (RR = 0.42; 95% CI: 1.15, 1.15) [34]. However, among MSM in Hong Kong SAR, there was very low-quality evidence that those in the HIVST group were more likely to report condomless anal intercourse (RR = 1.43: 95% CI: 0.98, 2.08) at 6-month follow-up than those in the standard HTS group.

### Social harm

One RCT [30] with very low-quality evidence reported on social harm following HIVST or standard facility-based HTS. In this trial, there were reports of a single harm in each group among two HIV-negative participants, 1/297 (0.34%) in the HIVST group and 1/303 (0.33%) in the control group, both relating to verbal and/or physical intimate-partner violence (IPV). In the HIVST group, the harm was not directly related to HIVST as the female participant reported violence occurred as a result of agreeing to participate in the study without consulting her husband. At enrolment neither participant reported experiencing IPV in the past 12 months, and the RCT used IPV screening tools and excluded women reporting risk of IPV [30].

## Discussion

Standard HTS approaches are essential and serve many people, but current approaches continue to miss a substantial number of people with HIV and those at high ongoing risk. This systematic review and meta-analysis finds there is moderate quality evidence that HIVST can increase the uptake of HIV testing and low-quality evidence that HIVST increases the frequency of HIV testing. This evidence is limited to MSM and male partners of pregnant and post-partum women in sub-Saharan Africa. However, these findings on increased uptake are consistent with the results of implementation studies from Kenya [13,35], Lesotho [17], Malawi [36,37], and Zimbabwe [38] which have been conducted among other populations known to have poor testing coverage, including men, young people and the households of people newly diagnosed with HIV, but do not directly compare with standard facility-based HTS.

Such increases in HIV testing uptake and frequency have important public health implications, if they can be achieved at a population level and reach those with undiagnosed HIV infection and at ongoing risk. As shown by two RCTs in this review [30,34] and reports from several other studies [12,15,31,36,39,40], increased testing due to HIVST can identify a greater or equivalent proportion of HIV infections as many existing HTS approaches. Sustained increases in HIV testing among men and other higher-risk populations, facilitated by HIVST, could identify a greater number of infections, and at an early stage in their infection [41], and result in earlier diagnosis and initiation of treatment and reduce HIV-related mortality. This is a particular priority for men, as they have greater HIV-related mortality than their female peers [42].

Limited information on linkage to care was identified in this review. Of the two RCTs reporting, one found that 72% (n = 396) of the male partners of women who received an HIVST kit said they accessed further testing to confirm their result [33]. This outcome, however, could not be directly compared with standard testing. In the other [30], while linkage following a reactive self-test appeared lower than those diagnosed in the standard group, few HIV-positive test results (n = 8) were reported. Additionally, this low level of linkage may be due to under-reporting and the possibility that some men already knew their HIV-positive status and were in care.

There are approaches following HIVST known to facilitate linkage to treatment, such as the offer of home-based ART initiation which resulted in a three-fold increase in linkage to care following HIVST in Malawi [43]. While results from a cluster-randomized trial in Malawi and a cohort study in Kenya suggest linkage to care following HIVST can be comparable to current national linkage rates [15,36], efforts to shorten the time between diagnosis and enrolment in care and improve overall linkage rates are needed. Further research is needed to identify ways to enhance linkage following HIVST; particularly for key populations, who may be less likely to link to services due to restrictive laws and policies.

Results from three RCTs [29,31,32,34] reporting on risk behaviours suggest HIVST did not increase risk-taking behaviour among MSM. While one RCT reported very low-quality evidence that HIVST could increase and having multiple sex partners among MSM in [31,32], results were not statistically significant. Additionally, data collected at baseline suggested high-risk MSM may be more likely to take up HIVST than standard HTS; and a sub-analysis among MSM who took up any testing across both arms found no effect on (RR = 0.81, 95% CI: 0.57, 1.75) and a minimal effect in reducing multiple male sex partners (RR = 0.72, 95% CI > 0.54, 0.95) [31,32]. Thus, while HIVST may not directly increase risk behaviours, there is some uncertainty and it is important that messages which reinforce the importance of using effective HIV prevention methods, such as condoms, are provided.

Only a single IPV event [30], which was not directly related to HIVST, was identified in this review of RCTs. Such findings are consistent with those reported by a review assessing harm resulting from self-testing for various conditions and diseases [44], an observational study in the United States among MSM [45], and a 2-year cluster-randomized trial [36] and parallel longitudinal qualitative study [24,46] in Malawi, which reported no cases of physical violence, self-harm or suicide and few cases of “coercion”.

In Malawi, the majority of those reporting “coercion” were men who also stated they were highly satisfied with HIVST (92%, 130/141) and would recommend it to others [36]. Qualitative findings from this same study also indicated that most users consider HIVST to be empowering, but some couples (n = 2/17) felt “pressure” to self-test by their partner and said serodiscordant HIVST results were challenging [24,46]. In contrast, a cohort study among 265 HIV-negative pregnant and post-partum women and female sex workers in Kenya reported two cases of IPV among post-partum women who distributed HIVST to their male partner and two cases of physical violence among female sex workers who distributed HIVST to their clients [15]. It is unclear if these cases were attributable to HIVST, as 41% of women in the study reported experiencing violence in the preceding 12 months [15]. These findings suggest that not all testing approaches are appropriate for all contexts, and caution is still needed in vulnerable populations. In order to guide safe HIVST implementation, programmes will need to consult populations at a risk of abuse. Additionally, HIVST may not be an appropriate or safe approach for all populations. It is important that information on where and how to access other HTS approaches, including community-based options, continues to be provided.

### Strengths and limitations

While other reviews on HIVST have assessed accuracy, feasibility and acceptability [18–21], this review is the first to directly compare HIVST to standard HTS and to systematically assess the effect of HIVST on uptake and frequency of testing, diagnosis of HIV-positive persons, linkage to care, risk behaviour and potential social harm. Additional strengths of this review include its ability to identify the latest evidence in both published and grey literature, its adherence to the PRISMA and Cochrane reporting standards and its consultation with global experts when defining the outcomes of interest to ensure finding would be relevant to the implementation and delivery of HTS (Appendix 2 (Supplemental material)).

The review and RCTs included, however, also have several limitations. Few studies which directly compared HIVST to standard HTS were identified in the review, and meta-analyses were only able to be performed among a small number of RCTs which had comparable outcomes. RCTs identified focused on male partners of women in antenatal or post-partum care and MSM, including sub-groups of recent and non-recent testers and young MSM. Other populations were not evaluated.

All RCTs compared HIVST to facility-based HTS. None compared HIVST to other community-based HTS approaches. Testing behaviour was assessed through self-report in all five RCTs and the potential for detection bias cannot be disregarded. However, self-reported data was validated with clinical records in two RCTs [29,32]. Two studies in this review were conference abstracts. However, we were able to contact all authors directly and obtain additional information, including full study protocols, which addressed some reporting gaps (Appendix 2 (Supplemental material)).

## Conclusions

This review found greater uptake of and frequency of HIV testing associated with HIVST compared to standard HTS. Risk-taking behaviour did not appear to increase due to HIVST, nor was HIVST associated with harm. Based on the findings of this review, and additional information reviewed at an expert meeting, WHO now recommends HIVST be offered as an additional HTS approach. Countries should make HIVST available and determine how to use this approach to fill gaps in testing coverage and reach those at risk who are not accessing existing HTS. Further assessment of different service delivery models and strategies to facilitate linkage, cost-effectiveness and the pathway to create supportive policies will be needed to maximize the potential of introducing HIVST.

## Notes

To access the supplementary material to this article please see Supplementary Files under Article Tools online.
